# A Solitary Benign Schwannoma of the Medial Dorsal Cutaneous Nerve of the Foot Masquerading as a Ganglion Cyst

**DOI:** 10.7759/cureus.16141

**Published:** 2021-07-03

**Authors:** Vivek Tiwari, Samir Dwidmuthe, Nisha Meshram

**Affiliations:** 1 Department of Orthopaedics, All India Institute of Medical Sciences Nagpur, Nagpur, IND; 2 Department of Pathology, All India Institute of Medical Sciences Nagpur, Nagpur, IND

**Keywords:** schwannoma, ganglion cyst, excisional biopsy, foot, nerve sheath

## Abstract

Schwannomas are rarely seen in the foot and ankle and mostly arise from the plantar aspect. Dorsal foot schwannomas are not commonly reported. We describe the case of a 25-year-old man who had a painful swelling on the dorsum of his right foot, which resembled a ganglion cyst on clinical examination and ultrasonography findings. Persistent symptoms prompted the need for an excisional biopsy, which revealed a benign schwannoma arising from the medial dorsal cutaneous nerve. Complete and careful excision relieved the symptoms without causing any distal neural deficit. Thus, schwannomas can mimic as a ganglion cyst. Excisional biopsy of such swellings, if symptomatic, helps in confirming the diagnosis, and provides a good functional outcome.

## Introduction

Peripheral nerve sheath tumors (PNSTs) are relatively rare tumors, arising from the sheath of cranial nerves, spinal nerves, or peripheral nerves. Among those, schwannomas, also known as neurilemmomas, are one of the most common benign neoplasms, arising from the Schwann cells [1]. Schwannomas are sporadic in the majority of the cases whereas multiple schwannomas are characteristically reported in neurofibromatosis type 2 (NF2) [2]. Bilateral vestibular schwannomas are almost diagnostic of NF2 [3]. The involvement of peripheral nerves by such tumors in the limbs is relatively rare, and mostly involves the flexor surfaces of the upper limbs. Schwannomas in the foot and ankle are infrequently described in the literature and mostly arise from the plantar aspect. Dorsal foot schwannomas are not commonly reported. Moreover, there have been only two cases of such tumors, which were found arising from the medial dorsal cutaneous nerve of the foot [4,5]. We report a third such benign schwannoma of the medial dorsal cutaneous nerve, which mimicked as a ganglion cyst based on the clinical characteristics and location.

## Case presentation

**Figure 1:**
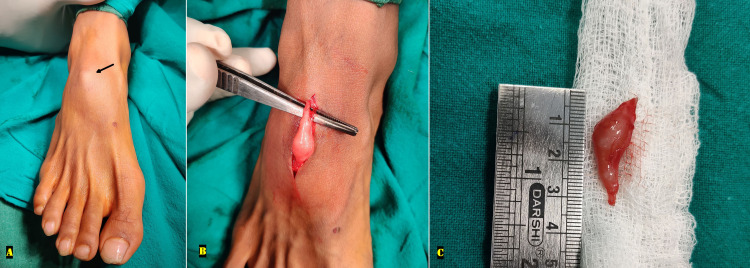
The clinical and intra-operative pictures of the foot swelling (A) A small superficial swelling was seen on the dorsum of the right foot (black arrow). (B, C) Incising the superficial fascia revealed a 3-cm long, smooth shiny bulbous mass attached to the medial dorsal cutaneous nerve, the swelling was carefully excised maintaining the continuity of the nerve fibers.

A 25-year-old male presented with complaints of a small painful swelling over the dorsum of the right foot for the past one year. There was no antecedent history of any trauma/ similar swellings elsewhere in the body neither was there any history of an increase in the size of the swelling. Aspiration was attempted from the swelling in another hospital but was not successful. Examination revealed a 2-cm, mobile, non-compressible, soft, tender swelling over the mid-dorsum of right mid-foot just proximal to the level of tarsometatarsal joints. Tinel’s sign was negative giving a clinical suspicion of a ganglion cyst. Visual analog score (VAS) was 6/10. There was no gait abnormality observed. Ultrasonography of the right foot demonstrated a soft tissue hypoechoic mass with posterior acoustic enhancement simulating a ganglion cyst; however, continuity of adjacent nerve fibers also gave a differential diagnosis of PNST. In view of the conflicting ultrasonographic and clinical findings, unavailability of MRI, and persistent symptoms, an excisional biopsy was planned. Upon giving incision in the superficial fascia, a smooth shiny bulbous mass was visualized in continuity with medial dorsal cutaneous nerve; careful excision of the mass was done maintaining the continuity of the nerve fibers. The clinical and intra-operative pictures of the swelling have been described in Figures 1A-1C.

**Figure 2:**
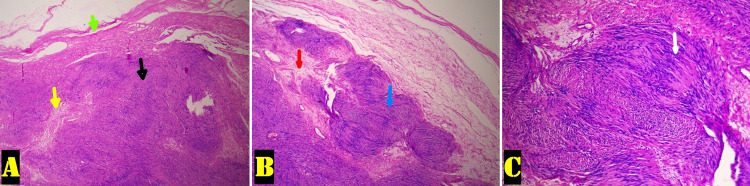
Histopathology images of the excised mass with hematoxylin and eosin staining (A) 4× magnification showing encapsulated mass (capsule - green arrow) with a mixture of hypocellular area (yellow arrow) and hypercellular area (black arrow). (B) 10× magnification showing Antoni A (blue arrow) and Antoni B (red arrow) areas. (C) 40× magnification showing Verocay bodies (white arrow).

The histopathology revealed an encapsulated mass showing an admix of myxoid hypocellular (Antoni B) and hypercellular areas (Antoni A) with nuclear palisading (Verocay bodies), confirming the diagnosis of benign schwannoma (Figures 2A-2C).

The patient’s symptoms were completely resolved after the surgery, and he resumed his normal activities from the next day. There was no neural deficit or any other complication noted after the excision. At the last follow-up of one year, the patient was pain-free (VAS 0) with no evidence of recurrence. Consent was obtained from the patient for the publication of this manuscript.

## Discussion

Schwannomas are a histological subtype of PNSTs, constituting 5% of all benign soft-tissue neoplasms [6]. Foot and ankle involvement with schwannoma is very rare with only around 60 cases reported to date [7]. Moreover, such tumors are extremely rarely seen on the dorsal aspect of the foot with only two cases of medial dorsal cutaneous nerve schwannoma described in the literature [4,5]. Our case was initially provisionally diagnosed as a ganglion cyst in view of the clinical and ultrasonographic profile. However, the lack of resolution of symptoms prompted us to have a second differential of schwannoma, for which an excisional biopsy was planned. In resource-limited settings like ours, MRI is often not feasible due to the lack of availability and economic reasons. In these scenarios, excisional biopsy of such swellings helps in arriving at the correct diagnosis, resolution of the symptoms, and differentiating them from ganglion cysts.

Peripheral nerve schwannomas usually present in the third-fourth decade with an equal predilection for male and female patients [7]. In the majority of the patients, such subcutaneous swellings are asymptomatic; however, these may sometimes cause localized pain and paraesthesia due to compression over the nerve fibers. Ultrasonography is the first-line investigation for these superficial swellings due to its noninvasive nature and low cost. The usual appearance of schwannoma is that of a homogenous, hypoechoic mass and may show its proximity to a nearby nerve. Sometimes, posterior acoustic enhancement and internal flow patterns may also be visible [8,9]. MRI can help in characterizing the tumors better, especially the deep-seated ones. The features on MRI of these tumors include hyperintense signals on T2-weighted images and iso-intense signals on T1-weighted images. Contrast MRI with gadolinium can help in differentiating schwannomas from ganglion cysts by identifying the nerve connection in the former cases. The asymptomatic swellings should be observed with time, as the risk of malignant transformation is very small even with the plexiform variety, with less than 15 cases reported; the risk increases in NF-2 and schwannomatosis [10]. Surgical excision is required in cases with large swellings and those with persistent symptoms [11]. The histopathological diagnostic findings include a peculiar division of compact hypercellular area (known as Antoni A), and loose hypocellular area (known as Antoni B) [2]. These tumors are reported to arise from the nerve sheath with splaying of the underlying nerve fibers [2]. Thus, careful surgical dissection should be done maintaining the continuity of the nerve fibers to prevent any post-operative neural deficit.

Ozdemir et al. described 196 primary bone and soft-tissue tumors involving foot and ankle at a tertiary center over 10 years, in which only two cases of benign schwannomas were reported [12]. In another large series of primary soft-tissue neoplasms of the foot from a cancer center in the US, schwannomas constituted only 2% (N=8) of all the tumors over a period of 15 years [13]. In a retrospective case series from Rizzoli Orthopaedic Institute Italy, the authors reported 14 schwannomas of foot among a total of 1,170 foot and ankle tumors over 18 years, with the majority of such schwannomas in the hindfoot [14]. Azevedo et al. reported five cases of schwannoma in a retrospective review of all primary musculoskeletal foot and ankle neoplasms treated at a musculoskeletal tumor referral center over 11 years [15]. In yet another large case series of foot and ankle tumors from a musculoskeletal tumor center in Germany, the authors described 11 schwannoma cases, out of a total of 413 tumors, over 18 years. None of those cases was reported in midfoot [16]. Our report describes a case of midfoot schwannoma arising from the medial dorsal cutaneous nerve, which masqueraded as a ganglion cyst. Jacobson et al. also reported a dorsal foot schwannoma; however, it was painless [4]. Capuzzi et al. described a similar case in a 60-year-old woman, which was managed with excisional biopsy with good results [5]. Thus, any subcutaneous swelling in the dorsal midfoot should be thoroughly investigated and schwannoma should be kept a differential in such cases. Excisional biopsy of these tumors helps in confirming the diagnosis and provides a good functional outcome.

## Conclusions

Schwannomas should be kept as a differential in all dorsal midfoot swellings. The clinical presentation of such swellings may mimic that of a ganglion cyst. Excisional biopsy of such swellings, if symptomatic, helps in confirming the diagnosis and provides a good functional outcome.
